# Spatial variations and determinants of iron containing foods consumption among 6–23 months old children in Ethiopia: spatial, and multilevel analysis

**DOI:** 10.1038/s41598-024-54959-0

**Published:** 2024-02-29

**Authors:** Bewuketu Terefe, Mahlet Moges Jembere, Birhanu Abie Mekonnen

**Affiliations:** 1 Department of Community Health Nursing, School of Nursing, College of Medicine and Health Sciences, University of Gondar, Po. Box: 196, Gondar, Ethiopia; 2 Department of Emergency and Critical Care Nursing, School of Nursing, College of Medicine and Health Sciences, University of Gondar, Gondar, Ethiopia; 3 Department of Pediatrics and Child Health, School of Medicine, College of Medicine and Health Sciences, University of Gondar, Gondar, Ethiopia

**Keywords:** Iron containing food, Spatial variation, Children, Determinants, Multilevel analysis, Ethiopia, Biomarkers, Medical research

## Abstract

Consuming foods high in iron benefits metabolic processes as well as the development of the neonatal and fetal brain. Despite the significance of eating foods high in iron for public health, Ethiopian practices are still limited when compared to the World Health Organization's (WHO) assessment of its consumption of such foods. This study used the Ethiopia Demographic and Health Survey (EDHS) to evaluate the consumption of iron-rich foods, regional clustering, and related characteristics among children aged 6–23 months. The information was taken from the typical EDHS 2019 dataset, which included a weighted sample of 1572 young children aged 6–23 months old in total. Utilizing Kuldorff's SaTScan version 9.6 software, spatial scan statistics were produced. Software from ArcGIS 10.8 was used to display the regional distribution of inadequate consumption of foods high in iron. Utilizing multilevel or mixed effects logistic regression analysis, the associated determinants for a healthy diet rich in foods containing iron were found. In the final model, a P-value of < 0.05 was announced as a statistical significance variable. Overall, in Ethiopia, children aged 6–23 months consumed iron-rich foods at a rate of 27.14% (95% CI 24.99–29.39). Poor intake of foods heavy in iron is concentrated in Ethiopia's regional states of Afar, a sizable portion of Amhara, Oromia, Tigray, Somali, Gambela, and SNNPS. Primary and secondary education (AOR = 1.73, CI 95%: 1.23, 2.41), and (AOR = 1.97,CI 95%: 1.25, 3.10), having ≥ 2 under five children, and current status of breastfeeding (AOR = 0.62 (CI 95%: 0.45, 0.84), and (AOR = 0.32, CI 95%: 0.23, 0.44), giving birth at health facilities (AOR = 1.51, CI 95%: 1.06, 2.13),being from Afar and Somali regions (AOR = 0.39, 95%: 0.17, 0.93), and (AOR = 0.26, CI 95%: 0.10, 0.69) have shown statistically significant association with the outcome variable respectively. In Ethiopia, providing high-iron meals and supplements to under-2-year-old children represents minimal, but persistent, public health expenses. Based on the identified determinants, the Ethiopian federal ministry of health and other stakeholders should pay special attention to the locations designated as hot spots for maternal and child health service enhancement to promote the consumption of iron-rich meals among children aged 6–23 months.

## Introduction

More than two billion individuals worldwide suffer from iron deficiency, with African children bearing the brunt of this problem^1,2^. Because micronutrient deficiencies are the most common nutritional deficiencies that lead to significant develop mental problems globally, iron deficiency anemia (IDA) continues to be a major public health concern, particularly for children and women in low and middle-income nations^3,4^. For immunological and nervous system development and cell proliferation, for the control of energy metabolism during exercise, and for the survival and virulence of many pathogens, iron is a crucial nutrient^5^. At a daily supplementation dose of 10–12.5 mg elemental iron, the World Health Organization (WHO) has consistently recommended oral iron supplementation as one of the therapies that can lower the prevalence of anemia^6^. According to WHO estimates, anemia affected 800 million women and young children globally in 2011; boosting iron consumption may reduce anemia by 42% in children and 50% in women^7^. African countries have been shown to have the highest burden of anemia among young children worldwide (62.3%)^8^. Nonetheless, children between the ages of 6 and 23 months are generally more susceptible to iron deficiency anemia^9^. Infants and young children are most affected by anemia because of their greater iron requirements associated to growth^10^.

Reports from EDHS 2016, and 2019 iron rich foods consumptions among 6–23 months old children was low 22%, and 24% only respectively^11,12^. From these figures anyone able to understand Ethiopia is still hitting her infants by iron rich food consumption scarcity with high rate of anemia.

Despite being high in protein, fat, and micronutrients, Ethiopians rarely eat foods made from animals because of the high cost. As a result, consumers in resource-constrained countries like Ethiopia lack both the physical access and financial means to buy fortified goods made from animal sources of food. Anemia and other micronutrient deficiencies may be caused by a low intake of foods derived from animals^13^. According to a study carried out in India, there is a significant inverse relationship between children's anemia and iron utilization^14^. Furthermore, the study demonstrated that preschool children with low iron intake have slower growth, lower immunity, and lower cognitive development^15^. Other factors such as age of the child wealth index, education level and health facility delivery were also among the reported in several studies^16,17^.

A crucial window of time is under the age of two that infant's chance for development and growth. This crucial time could have an impact on how the kids grow, learn, and develop in the future. Later^8,18^. Therefore, a child needs during this time proper nutrition is essential for development in all areas, including growth, physical maturity, and mental and emotional health. Despite the aforementioned facts, there is no study that children consume iron-rich foods in Ethiopia between 6 and 23 months old. This's goal is as a result. The purpose of the study was to evaluate the geographic distribution of iron-rich areas, consumption of food and its contributing factors for youngsters in Ethiopia between 6 and 23 months old.

## Methods and materials

### Study design, period and setting

A community-based cross-sectional study was employed in Ethiopia from March to June 2019^12,19^. Ethiopia is a country in the Horn of Africa found in East Africa (3°–14°N and 33°–48°E) with nine regional states (Afar, Amhara, Benishangul-Gumuz, Gambella, Harari, Oromia, Somali, Southern Nations, Nationalities, and People's Region (SNNP) and Tigray) and two city administrations (Addis Ababa and Dire Dawa). It has 68 zones, 817 districts, and 16,253 kebeles (lowest administrative units of a country). It has a population of over 110 million. Of which, 39.81% of the people are less than 14 years with a 1:1 sex ratio to the general population. The country also has a death rate of 5.8/1000, 22.2% of urbanization, with a very high degree of major infectious diseases^20,21^. The current Ethiopian Demographic and Health Survey 2019 (EDHS 2019) was used by the study to analyses the regional distribution and factors influencing young children iron intake in Ethiopia. Data on a variety of elements of women's health and welfare, including problems with interpersonal violence, are collected by the Demographic and Health Survey (DHS). The survey used a representative sample of 1572 weighted young children aged 6–23 months from across the country. The DHS website, http://www.dhsprogram.com, hosted the data that we needed for our study, and we downloaded it once our request for access was accepted and downloading was permitted.

### Source and study population

The study population consisted of all sampled living or selected children aged 6–23 months preceding 5 years of the survey period in the chosen Enumeration Areas (EAs) in the country, while the source population consisted of all living children in Ethiopia who were 6–23 months prior to the survey period. Children whose outcome variable data was incomplete were not included in the analysis. In the event that a single household has two or more children, the youngest child will be chosen for an interview based on the aim of the study. Furthermore, if the children are twin other techniques such as lottery method might be applied by the data collector.

### Sample size determination and sampling methods

The EDHS sample was divided into two groups before being chosen. 21 sampling strata were produced after stratifying each region into urban and rural areas. Enumeration Areas (EAs) samples were chosen independently in each stratum over the course of two stages. By classifying the sampling frame within each sampling stratum before sample selection, according to administrative units at various levels, and using a probability proportional to size selection at the first stage of sampling, implicit stratification and proportional allocation were achieved at each lower administrative level.

In the first stage, 305 EAs (93 in urban areas and 212 in rural regions) were chosen independently in each sampling stratum with a probability proportionate to the size of the EA. In all chosen EAs, a household listing operation was conducted from January through April 2019. The lists of households that were produced served as a sampling frame for choosing households in the subsequent stage. With more than 300 homes, several of the EAs chosen for the 2019 Ethiopian Demographic and Health Survey (EDHS) were sizable. Each significant EA chosen for the 2019 EDHS was split to reduce the burden of household listing. The probability was proportionate to the segment size, and there was only one segment chosen for the survey. A 2019 EDHS cluster is either an EA or a component of an EA; household listing was only done in the chosen segment. From the newly constructed household listing, a fixed number of 30 households per cluster were chosen in the second round of selection with an equal likelihood of systematic selection^19^.

### Study variables

Iron rich foods intake in infants 6–23 months was took an outcome variable. During the survey, their mother was asked questions about their living children 6–23 months who consumed foods rich in iron at any time in 24 h preceding the interview any of egg, meat, liver, heart, other organs and child fish or shellfish^22^. When a child between the ages of 6 and 23 months consumes at least one iron-rich food item from the list above at any point in the 24 h leading up to the interview, it is considered good consumption of iron-rich foods; when no iron-rich food is consumed in the 24 h leading up to the interview, it is considered poor consumption of iron-rich foods^22^. Then, the outcome variable is categorized with “Yes = 1” and “No = 0”. In this study both individual and community level factors have been considered. The independent variables were sex of the household head, age of mother, women education levels, marital status, family size, wealth index, current breastfeeding status, sex of the child, age of the child, plurality of the child, birth order, antenatal visits, place of delivery, and number of under five children were included as an individual level factor. Regarding community level factors types of residence, region, community level women education level, and community level poverty were included in the analysis.

### Operational definitions for community level variables

All community level components were calculated using their aggregate values because they could not be observed or documented during the survey. Although the method was the same as in other literatures, each of them was calculated according to the value of each individual variable. In this study, a community level factor was defined as a collection of households that shared a primary sample unit or cluster in the dataset. mixing elements at the individual level to create variables at the community level. The community variables included region, place of residence, community women's education (% of women with primary or post-primary education), and community poverty (proportion of households in the community who were poor). To make the results easier to understand, the continuous community-level variables were further divided into low and high categories using the mean/median value based on their distribution^23–25^.

#### Community women education

Based on the typical distributions of women’s educational levels in the community, this is the sum of the educational levels of all interviewed women from every household. The ratio of women from each household in the community with a secondary education or higher was classified as low if it was below the median (0–8.39%) and high if it was above (8.4–100%). The number 8.4% was the median value.

#### Community poverty

The same process is used to get this variable from each household's wealth index. In a specific community, it was considered high if the ratio of households from the two quintiles with the lowest levels of wealth was 35.72–100% and low if it was 0–35.71%. 35.72% was the median value.

### Data collection tool, quality control, and procedures

In the DHS, a pre-test was conducted before data collection, a debriefing session with pre-test field workers was held, and changes to the questionnaires were made as necessary. The DHS guidance has more details about the data collection process^26^. Every 5 years, the DHS collects data by trained professional data collectors that are nationally representative and reflect the demographic and health challenges unique to each nation using five different surveys. These included questionnaires for households, women, and men, biomarkers, and health institutions. The kids record questionnaire used in this study was used to ascertain the country iron containing foods consumption, and contributing factors among 6–23 months old young children^22,26^.

### Data management processes, model building, and analysis

The STATA-formatted standard DHS data from the dataset were downloaded, cleaned, integrated, converted, and appended to create favorable variables for the analysis. While STATA 17 software was used for analytic statistics, Microsoft Excel was used to perform descriptive analyses and to aggregate some community level variables. Software’s like ArcGIS 10.8 and SaTscan were also applied for the spatial parts of analyses.

Children between the ages of 6 and 23 months were embedded within a cluster in the hierarchical DHS data. The assumptions of independence and equal variance may be broken by this. The result was the fitting of a multilevel binary logistic regression model. For multi-level analysis, four models were fitted. The first was the null model (Model 1), which had no exposure factors and was used to examine the cluster's variability. Individual-level factors and community-level variables are present in the second (Model 2) and third (Model 3) multilevel models, respectively. The prevalence of iron consumption among those 6 to 23-month-old young children was fitted simultaneously with individual and community level variables in the fourth model (Model 4).

$$ {\text{Log }}\left( {\uppi {\text{ij}} \div {1} - \, \pi {\text{ij}}} \right)  =  \upbeta 0 + \upbeta {\text{1xij }} + \upbeta {\text{2xij }} + \cdots {\text{uj }} + {\text{eij}} $$

where πij is the likelihood that no iron will be consumed, and ij is the likelihood that it will. When none of the explanatory factors are present, the influence on feeding iron intake is represented by the intercept, or β0. The variables at the individual and community levels for the ith person in group j are βxij, respectively. Also, because the β's are fixed coefficients, a rise in X can result in an increase in the risk of ingesting iron by an additional ß unit. The UK demonstrates the jth community's random effect—the influence of the community on the mother's decision to supply iron intake. Assuming that each community has a unique intercept (β0) and fixed coefficient (β), the clustered nature of the data as well as between and between community variances were taken into consideration^27,28^.

The likelihood test was used to compare the models, and model 4 was found to have the greatest value and be the best fit. All variables had VIF values less than 10, and the mean VIF value of the final model was 1.50, which was utilized to identify multicollinearity. Crude Odds Ratio (COR) and Adjusted Odds Ratio were used to measure the relationship between the dependent and independent variables (AOR). For the final model, factors having a p-value of less than 0.2 in COR have been chosen as contenders. Adjusted odds ratios and 95% confidence intervals with a p-value of 0.05 were used to determine the strength of associations between dependent and independent factors. The Median Odds Ratio (MOR), which is defined as the median value of the odds ratio between the area at the lowest risk and the highest risk when randomly choosing two clusters, was used to assess the measure of variance. MOR = e0.95√VA or, MOR = exp. [√ (2 × VA) × 0.6745], where; VA is the area level variance^27,28^. The Proportional Change in Variance (PCV reveals the variation in iron consumption among children 6–23 months explained by factors. The PCV is calculated as = $\frac{Vnull-VA}{Vnull}$*100. Where: Vnull is the initial model's variance and VA is the model's variance with additional terms. Also, the Intra Class Correlation Coefficient (ICC), a measurement of the variation in iron consumption between clusters, is computed as; ICC = VA ÷ VA + 3.29 ∗ 100%, where; VA = area/cluster level variance^27,28^.

### Consent to participate and ethical approval

Ethical approval and a letter of authorization were acquired on the website http://www.dhsprogram.com, and the DHS programme was given permission through email to access the data for this study. This study used publicly available data that was entirely devoid of any personally identifiable information. The study used secondary data from the EDHS. The EDHS authorities ethically handled our concerns regarding informed consent, confidentiality, anonymity, and privacy of the study sample, and we did not change or use the data in any other way. The general public and patients were both left out of this research.

## Results

### Sociodemographic characteristics of the study participants

A total of 1572 weighted mothers with their infants in Ethiopia were included in this study. About half of them 7849 (49.89%) of the study participants were with an estimated age range of 25–34 years old, however most of them 1354 (86.12%), and 1485 (94.45%) were male household heads and married women. Regarding education and wealth index of the house about 639 (40.68%), and 343 (21.83%) were mothers with no formal education enrollment and richest respectively. Large amount of the study participants came from large family size^5–10^, and Oromia regions with a figure of 948 (60.32%), and 602 (38.31%) respectively. Similarly, about 1138 (72.38%), 994 (63.26%), and 913 (58.06%) of the study participants were came from rural areas, low community poverty and high women education communities respectively (Table 1).

**Table 1:** Socio-demographic characteristics of the study participants in determinants of iron supplements among infants 6–23 months in Ethiopia: based on 2019 EDHS (weighted n = 1572).

Variables	Iron foods supplementation		Total, n (%)
	No, n (%)	Yes, n (%)	
Age of women (years)			
15–24	364 (72.05)	142 (27.95)	506 (32.17)
25–34	570 (72.71)	214 (27.29)	7849 (49.89)
35–49	211 (74.74)	71 (25.26)	282 (17.94)
Sex of household head			
Male	980 (72.39)	374 (27.61)	1354 (86.12)
Female	165 (75.75)	53 (24.25)	218 (13.88)
Educational attainment of women			
No formal education	575 (80.65)	138 (19.35)	713 (45.32)
Primary	436 (68.20)	203 (31.80)	639 (40.68)
Secondary and above	135 (11.76)	85 (20.02)	220 (14.00)
Marital status of the mother			
Not married	61 (70.08)	26 (29.92)	87 (5.55)
Married	1,084 (73.02)	401 (26.98)	1,485 (94.45)
Household family size			
1–4	395 (67.18)	193 (32.82)	588 (37.40)
5–10	719 (75.80)	230 (24.20)	948 (60.32)
= >11	32 (88.39)	4 (11.61)	36 (2.28)
Wealth index			
Poorest	265 (83.83)	51 (16.17)	316 (20.12)
Poorer	239 (71.02)	97 (28.98)	336 (21.40)
Middle	244 (81.55)	55 (18.45)	299 (19.01)
Richer	178 (64.00)	100 (36.00)	278 (17.64)
Richest	220 (64.14)	123 (35.86)	343 (21.83)
Region			
Tigray	70 (62.96)	41 (37.04)	111 (7.04)
Afar	20 (87.45)	3 (12.55)	23 (1.44)
Amhara	259 (76.53)	80 (23.47)	339 (21.54)
Oromia	432 (71.66)	171 (28.34)	602 (38.31)
Somali	88 (91.89)	8 (8.11)	96 (6.12)
Benishangul-Gumuz	13 (68.49)	6 (31.51)	19 (1.18)
SNNP	220 (70.83)	91 (29.17)	311 (19.79)
Gambela	5 (75.13)	2 (24.87)	7 (0.46)
Harari	3 (64.34)	2 (35.66)	5 (0.28)
Addis Ababa	29 (57.31)	22 (42.69)	51 (3.24)
Dire Dawa	7 (66.63)	4 (33.37)	10 (0.62)
Residence			
Urban	289 (66.47)	146 (33.53)	435 (27.62)
Rural	857 (75.30)	281 (24.70)	1138 (72.38)
Community level poverty			
Low	679 (68.31)	315 (31.69)	994 (63.26)
High	466 (80.69)	112 (19.31)	578 (36.74)
Community women education			
Low	535 (81.15)	124 (18.85)	659 (41.94)
High	610 (66.87)	302 (33.13)	913 (58.06)

### Children related descriptive characteristics

In this study most 1065 (67.77%) of infants were found between 6 and 23 months old, however, they were nearly similar regarding the sex proportion. Nearly all of them 1537 (97.78%) were single birth, and about 626 (39.81%) were classified as fourth and above birth orders. Most mothers 1279 (81.39%) were breast feeder, have at least one ANC visit 1197 (76.13%), and about 876 (55.71%) of them gave birth at health facilities (Table 2).

**Table 2:** Child related characteristics and health service utilization-related factors of iron supplements among infants 6–23 months old in Ethiopia: based on 2019 EDHS (weighted n = 1572).

Variables	Iron foods supplementation		Total, n (%)
	No, n (%)	Yes, n (%)	
Age of the child (months)			
6–11	388 (76.51)	119 (23.49)	507 (32.23)
12–23	758 (71.12)	307 (28.88)	1065 (67.77)
Sex of the child			
Male	600 (73.09)	221 (26.91)	821 (52.20)
Female	545 (72.60)	206 (27.40)	751 (47.80)
Plurality			
Single	1,119 (72.82)	418 (27.18)	1,537 (97.78)
Multiple	26 (74.69)	9 (25.31)	35 (2.22)
Order of birth			
First	273 (69.79)	118 (30.21)	391 (24.90)
2–3rd	395 (71.14)	160 (28.86)	555 (35.29)
4th and above	478 (76.30)	148 (23.70)	626 (39.81)
Number of under five children			
0–1	481 (68.89)	217 (31.11)	698 (44.37)
2 and above	665 (76.03)	209 (23.97)	874 (55.63)
Breast feeding status			
No	184 (62.75)	109 (37.25)	293 (18.61)
Yes	962 (75.17)	318 (24.83)	1,279 (81.39)
ANC visits			
No	296 (78.80)	80 (21.20)	375 (23.87)
At least one	850 (71.00)	347 (29.00)	1,197 (76.13)
Place of delivery			
Home	530 (76.06)	167 (23.94)	696 (44.29)
Health facility	616 (70.32)	260 (29.68)	876 (55.71)

### Iron containing foods consumption among children aged 6–23 months in Ethiopia by regions

**Figure 1:**
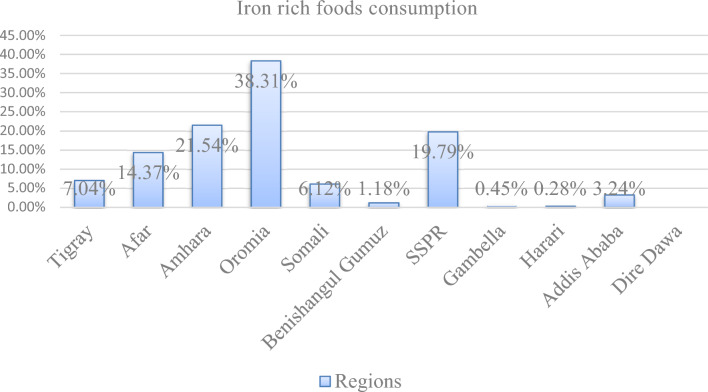
Shows the weighted Iron rich foods consumption coverage by regions among children aged 6–23 months in Ethiopia.

According to the following figure data the highest proportion of iron rich foods consumption was seen in Oromia region by 38.31%, and the lowest was from Gambella and Harari regions (Fig. 1).

### Spatial distribution of iron rich food taking coverage

**Figure 2:**
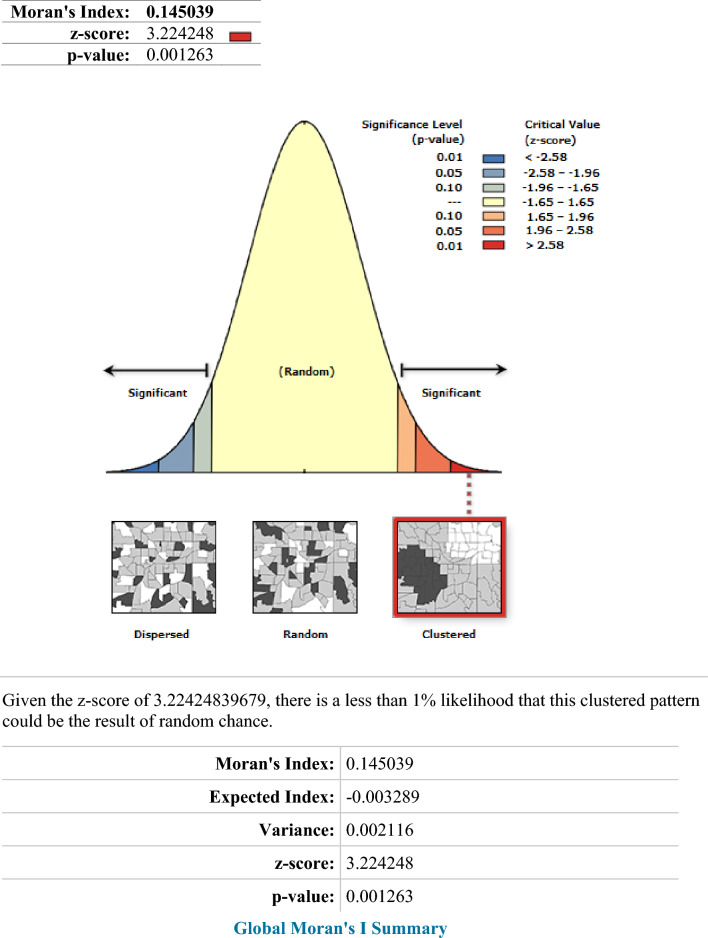
ArcGIS version 10.8 (https://www.arcgis.com/index.html) results for spatial autocorrelation report analysis of Iron containing foods intake among children aged 6–23 months in Ethiopia.

**Figure 3:**
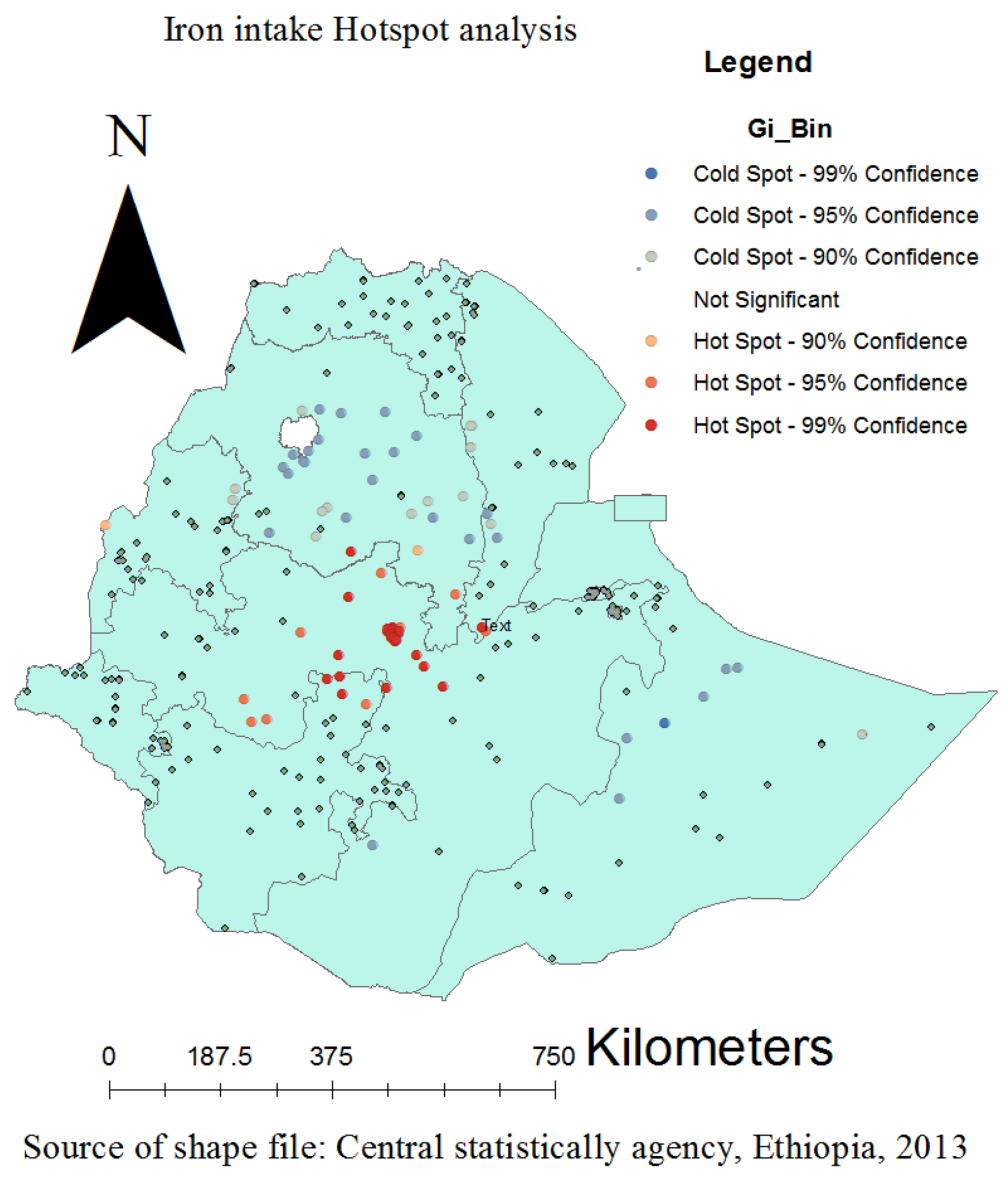
ArcGIS version 10.8 (https://www.arcgis.com/index.html) results for spatial distribution of Iron containing foods intake among children aged 6–23 months in Ethiopia, EDHS.

According to this study, Ethiopia had a spatially clustered pattern of Iron containing foods intake coverage with a Global Moran's I of 0.145039 (P-value of 0.001263). Over the research area, a distribution of taking of iron rich foods consumption coverage with high rates was seen. On the right and left sides of each panel, there were automatically produced keys. Given the z-score of 3.224, the chances that this clustered pattern is accidental is less than 1%. The terminal tails' vivid red and yellow coloring denotes a higher level of significance (Fig. 2). At the regional level, spatial clustering of taking of iron rich foods consumption was discovered. Just about 426 (27.14%) of the weighted 1572 infants and young children aged 6–23 months surveyed in 2019 had consumed iron rich foods coverage. Addis Ababa, North Shewa of Amhara region, and central Oromia had the greatest taking of iron rich foods utilization rates, whereas Harari, Somali, most Amhara, Tigray, Afar, SNNPRs, and Gambella regions had revealed the lowest iron rich food consumption rates (Fig. 3).

### Spatial interpolation of iron rich foods consumption in Ethiopia

**Figure 4:**
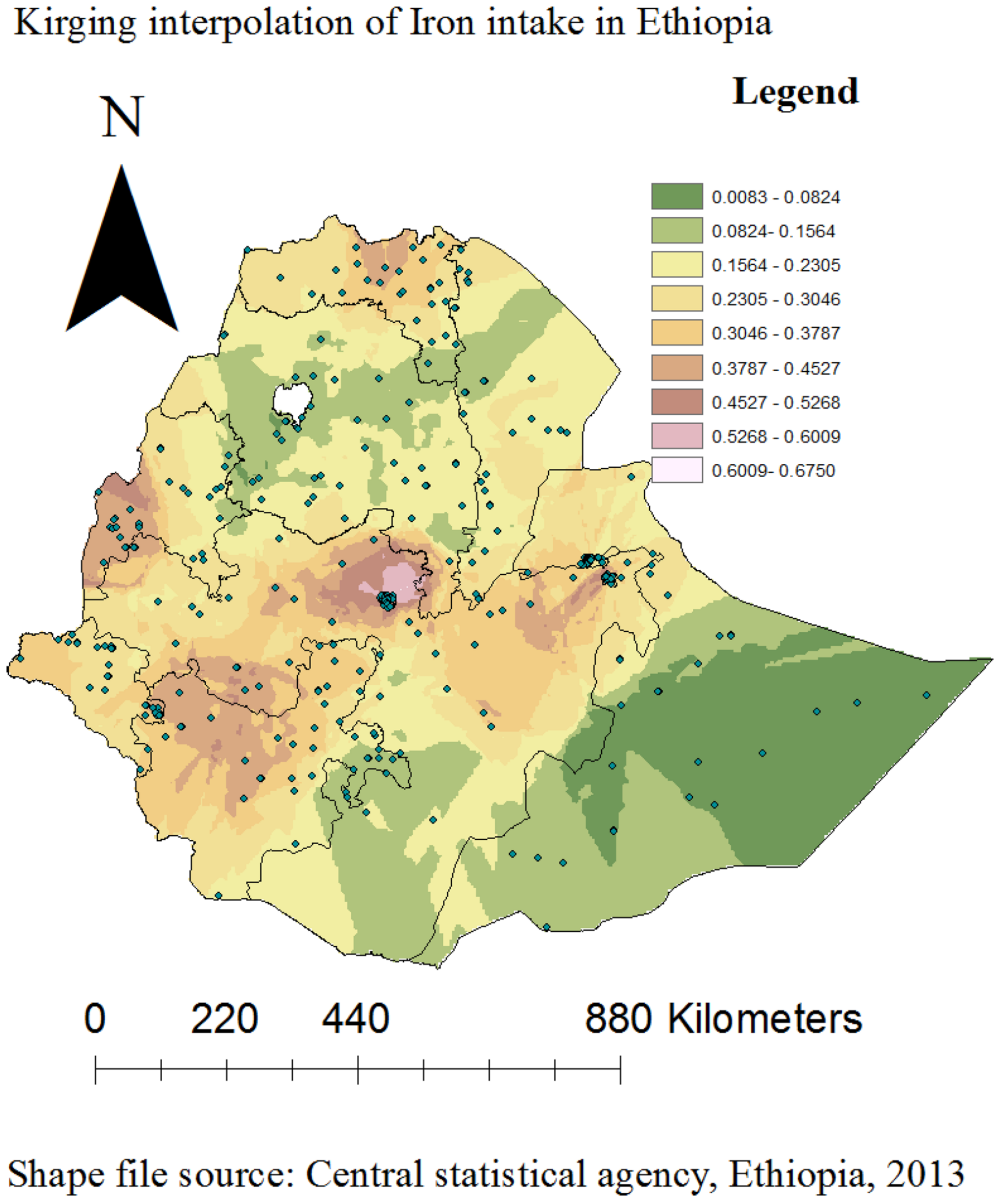
ArcGIS version 10.8 (https://www.arcgis.com/index.html) results for interpolation predictions of Iron rich foods consumption in Ethiopian across regions, Ethiopia.

The iron rich foods consumption was found in various portions of Ethiopia, including practically Somali, large parts of the Amhara, southern Oromia and central Afar regions had shown higher rates of consumptions. Applying the Kriging interpolation approach produces high coverage predictions of iron rich foods taking. On the other hand, the remaining regions had low coverage projection (Fig. 4).

### Spatial SaTScan analysis of iron containing foods intake (Bernoulli based model)

**Figure 5:**
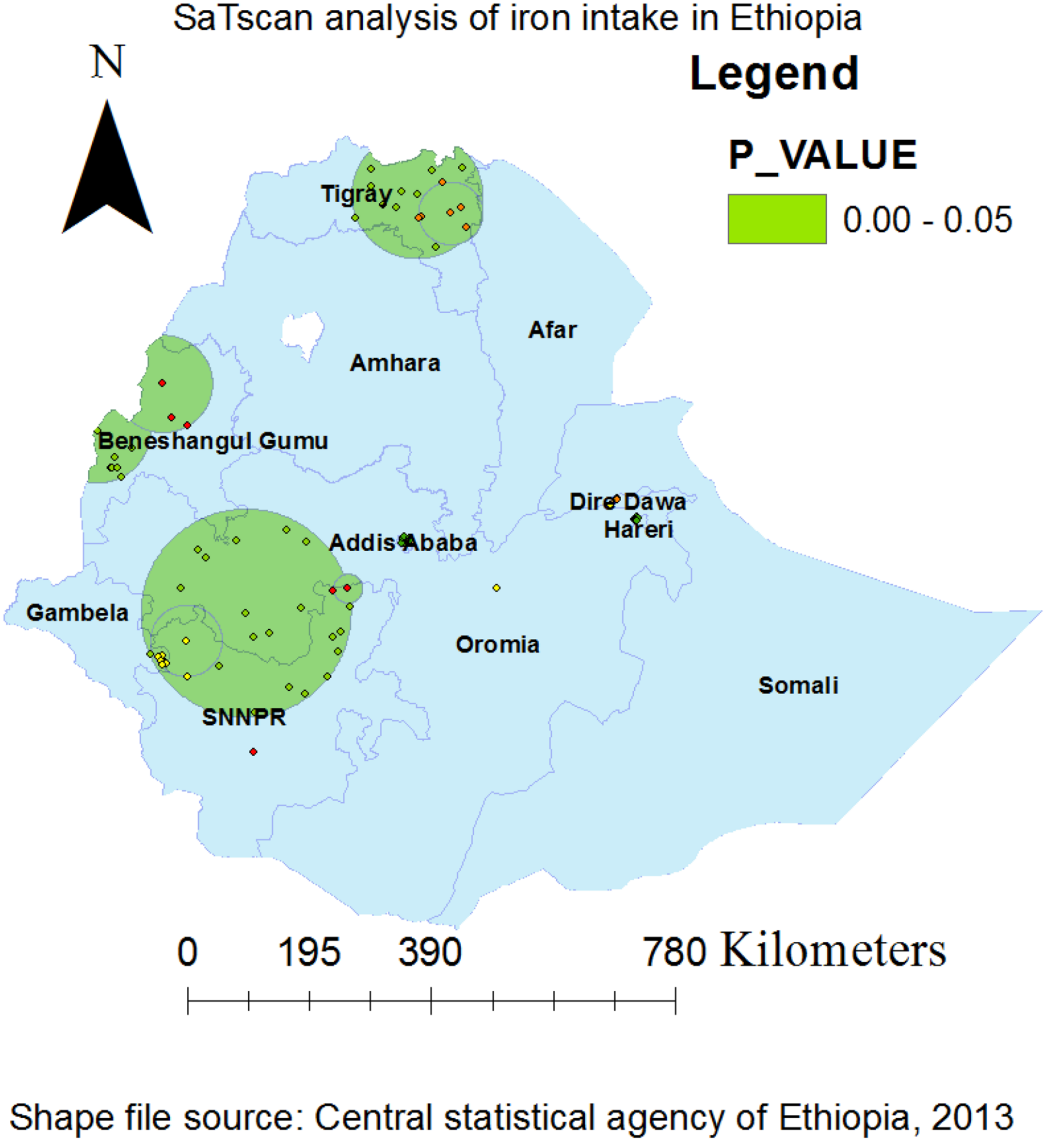
Kuldorff's SaTScan version 9.6 software (https://www.satscan.org/) results for primary and secondary clusters of Iron containing foods consumption among children aged 6–23 months across regions in Ethiopia.

Both the primary and secondary taking of iron containing foods clusters that are most likely were found. Spatial sat scan statistics for EDHS 2019 revealed a total of both high- and low-performing spatial clusters of iron rich foods consumption among infant and young children aged 6–23 months in Ethiopia. Only 25 significant clusters were found using the spatial scan analysis on these, with the primary, and secondary clusters each consisting of 13 clusters, and 12 clusters respectively. The primary sat scan window was found at (9.329504 N, 42.103751 E) with a radius value of 4.01 km in the Tigray, Benishangul-Gumuz, Addis Ababa city and central Oromia (zones of Shewa districts) regions. This cluster window has a relative risk of 2.30, a log likelihood ratio (LLR) of 11.98, and a p-value of 0.002 with 52 population and 730 cases. In this scanning window, the practice of iron rich foods consumption among children implementation coverage was 2.30 times higher to use it than other areas outside the window. The most statistically significant iron rich foods consumption coverage spatial windows are denoted by vivid green circles in the SaTscan analysis map. The most likely cluster, which SaT Scan also identified as the secondary cluster, did not physically overlap with it. With a coordinate (8.995815 N, 38.793907 E), and distance of 7.13 km, Ethiopia's central Oromia region at (8.995815 N, 38.793907 E) is where the secondary SaTScan circular window is most frequently seen. About 37 infants and young children make up this cluster as well, and 23 cases with a relative risk of 2.46 and a likelihood of it of 10.89 with a p-value of 0.0053 are among them. According to statistics, this cluster indicates that iron rich foods consumption coverage inside the circular window is 2.46 times more likely to be available than it is for infants and young children aged 6–23 months outside the window (Table 3 and Fig. 5).

**Table 3:** Significant spatial clusters with high-rate iron rich foods taking coverage among children aged 6–23 months in Ethiopia, EDHS, 2019.

Cluster	Enumeration areas (Cluster detected)	Coordinates (radius)	Population	Cases	RR	LLR	P-value
1	232, 231, 246, 237, 240, 238, 235, 253, 243, 242, 233, 239, 234	(9.329504 N, 42.103751 E)/4.01 km	52	32	2.30	11.99	0.002
2	273, 264, 267, 270, 271, 276, 263, 275, 265, 266, 268, 272	(8.995815 N, 38.793907 E)/7.13 km	37	23	2.46	10.89	0.005
3	97, 96, 91, 95, 195, 194, 120, 94, 93, 92, 201, 204, 179, 174, 98,221, 87, 223, 222, 177, 227, 226, 224, 196, 180, 191, 189, 176, 225, 171	(7.964041 N, 36.490345 E)/166.45 km	120	49	1.64	6.70	0.226
4	11, 12, 14, 13, 2, 16, 10, 7, 17, 1, 3, 6, 15, 23, 25, 9	13.985245 N, 38.954809 E)/101.70 km	90	38	1.68	5.85	0.348
5	146, 157, 149, 147, 152, 153, 154	(10.589922 N, 34.352538 E)/83.30 km	34	18	2.08	5.65	0.372
6	102	(8.313592 N, 40.103390 E)/0 km	4	4	3.86	5.39	0.751
7	194, 221, 222, 223, 226, 227, 224, 201	(7.564452 N, 35.630124 E)/57.01 km	27	15	2.17	5.34	0.586
8	305	(9.521566 N, 41.739325 E)/0 km	9	7	3.01	5.28	0.605
9	282,284	(9.614701 N, 41.829121 E)/1.54 km	14	9	2.50	4.53	0.775
10	17, 3, 25, 14, 16, 2	(13.721325 N, 39.439019 E)/50.24 km	40	19	1.86	4.32	0.849
11	171,174	(8.318202 N, 37.960553 E)/23.09 km	13	8	2.38	3.63	0.970
12	192	(5.967423 N, 36.604504 E)/0 km	5	4	3.09	3.19	0.995
13	159, 160, 158	(11.267438 N, 35.292874 E)/77.95 km	19	10	2.04	3.05	0.998

### The highlights of random parameters and model comparison

This study used multilevel logistic regression to fit the model. Hence, four fitted models including the null model, model one, model two, and model three were used to show the effect of fixed and random. In null model showed a significant variance in the probability of not taking iron rich foods among infants and young children in Ethiopia (community level variance = 50.41, p < 0.001). As implied by the intra-cluster correlation coefficient (ICC) in the empty model, region differences would be 17.89% of the variation in infants and young children. The null model's ICC value of 17.89% indicates that variations between clusters account for the majority of the overall variability in of iron rich foods consumption, with individual differences accounting for the remaining 82.11%. Furthermore, the median odds ratio (MOR) was 2.24 (1.79, 2.70), this can be interpreted as when infants and young children go from low to high taking of iron rich foods utilization area, the likelihood of consumption of iron containing foods was 2.24 times higher. The PCV indicates both community-level and individual-level variables explained by its value of the national variation observed in an empty model. The combined individual and community level model (model III), which is the ideal model for the nature of this data, was the model that best suited the data since it had the lowest deviation value in comparison to other models (Table 4).

**Table 4:** Multilevel fixed effect (individual and community level) analysis of factors associated with iron containing foods intakes among infants 6–23 months old in Ethiopia.

Independent variables	Null model	Model I	Model II	Model III
		AOR [95% CI]	AOR [95% CI]	AOR [95% CI]
Age of women (years)				
15–24		1		1
25–34		1.12 (0.79, 1.60)		1.03 (0.72, 1.48)
35–49		1.32 (0.79, 2.20)		1.19 (0.71, 2.00)
Sex of household head				
Male		1		1
Female		0.71 (0.51, 1.00)		0.75 (0.53, 1.07)
Educational level of women				
No formal education		1		1
Primary		1.90 (1.36, 2.63)		1.73 (1.23, 2.41)*
Secondary and above		2.19 (1.41, 3.40)		1.97 (1.25, 3.10)*
Household family size				
1–4		1		1
5–10		0.95 (0.68, 1.32)		0.96 (0.69, 1.33)
= > 11		0.83 (0.37, 1.86)		0.91 (0.40, 2.04)
Wealth index				
Poorest		1		1
Poorer		1.94 (1.25, 3.00)		1.37 (0.87, 2.17)
Middle		1.26 (0.78, 2.04)		0.84 (0.50, 1.42)
Richer		2.07 (1.28, 3.35)		1.24 (0.72, 2.14)
Richest		1.83 (1.14, 2.94)		0.84 (0.43, 1.64)
Age of the child (months)				
6–11		1		1
12–23		1.17 (0.88, 1.55)		1.14 (0.86, 1.52)
Plurality				
Single		1		1
Multiple		2.00 (0.91, 4.38)		2.13 (0.97, 4.66)
Birth order				
First		1		1
2–3rd		0.88 (0.59, 1.31)		0.89 (0.59, 1.33)
4th and above		0.92 (0.54, 1.56)		0.89 (0.52, 1.53)
Number of under five children				
0–1		1		1
2 and above		0.60 (0.44, 0.81)		0.62 (0.45, 0.84)*
Breast feeding status				
No		1		1
Yes		0.35 (0.26, 0.47)		0.32 (0.23, 0.44)*
ANC visits				
No		1		1
At least one		0.89 (0.61, 1.30)		0.82 (0.56, 1.21)
Place of delivery				
Home		1		1
Health facility		1.68 (1.19, 2.37)		1.51 (1.06, 2.13)*
Regions				
Tigray			0.89 (0.43, 1.86)	1.23 (0.57, 2.67)
Afar			0.31 (0.14, 0.69)	0.39 (0.17, 0.93)*
Amhara			0.44 (0.21, 0.92)	0.59 (0.27, 1.31)
Oromia			0.81 (0.40, 1.61)	0.96 (0.45, 2.04)
Somali			0.23 (0.09, 0.54)	0.26 (0.10, 0.69)*
Benishangul-Gumuz			0.97 (0.46, 2.01)	1.12 (0.50, 2.48)
SNNPs			0.76 (0.37, 1.53)	0.85 (0.39, 1.82)
Gambela			0.68 (0.32, 1.44)	0.78 (0.35, 1.77)
Harari			1.05 (0.53, 2.10)	1.28 (0.61, 2.68)
Addis Ababa			1	1
Dire Dawa			0.74 (0.37, 1.50)	0.81 (0.39, 1.70)
Residence				
Urban			1	1
Rural			0.92 (0.61, 1.39)	0.87 (0.53, 1.44)
Community level poverty				
Low			1	1
High			0.68 (0.47, 0.98)	0.72 (0.47, 1.11)
Community women education				
Low			1	1
High			1.63 (1.15, 2.33)	1.34 (0.91, 1.96)
Random parameters and model comparison				
Community-level variance	0.72 (0.43, 1.19)	0.43 (0.21, 0.86)	0.33 (0.15, 0.72)	0.35 (0.16, 0.78)
ICC (%)	17.89	11.59	9.13	9.06
MOR (95% CI)	2.24 (1.79, 2.70)	1.87 (1.46, 2.28)	1.73 (1.36, 2.10)	1.76 (1.36, 2.16)
PCV (%)	Reference	0.41	0.54	0.52
Log-likelihood (LLR)	− 898.03	− 813.02	− 857.05	− 796.24
DIC (−2LLR)	1796.06	1626.04	1714.10	1,592.48
AIC	1800.07	1668.05	1744.09	1660.47
BIC	1810.82	1780.96	1824.74	1843.27

*Indicates continuous variable and significance level at p-value < 0.05 in the final regression model.

### Factors associated with iron intake among under two young children in Ethiopia

Determinants such as women education levels, number of under five children in the family, health facility delivery, being breast feeder, and regions were statistically significant in the multilevel multivariable logistic regression model in of Iron foods intake among infants in Ethiopia. Mothers who have accomplished their primary and higher educational status have shown (AOR = 1.73 (CI 95% 1.23, 2.41)), and (AOR = 1.97 (CI 95%: 1.25, 3.10)) times to provide iron rich foods to their children compared to mothers without formal education respectively. However, mothers who have two and more than two under five children, and currently breast feeders have shown a less likely probability of feeding Iron rich foods to their children by the odds of (AOR = 0.62 (CI 95%: 0.45, 0.84)), and (AOR = 0.32 (CI 95%: 0.23, 0.44)) respectively compared to mother who have a maximum of one child and currently not breast feeding. Those mothers who have given their birth in the health facilities have shown (AOR = 1.51 (CI 95%: 1.06, 2.13)) times more chance of providing Iron rich foods to their children compared to mother who delivered in other than health facilities. Regarding to regions compared to Addis Ababa, Afar and Somali regions have revealed much less probability of providing Iron rich foods to infants by the odds of (AOR = 0.39(95%:0.17,0.93)), and (AOR = 0.26 (CI 95%: 0.10, 0.69)) respectively (Table 4).

## Discussion

Evidences from the three consecutives EDHS of 2011, 2016 and 2019 have revealed that anemia among infants accounts large proportion with a low rate of iron rich food consumption not more than 24%^11,12,29^, however, the WHO's guidelines recommended for infants and young children (6–23 months) living in areas where anemia prevalence is 40% or greater in this age group, daily iron supplementation is advised as a public health intervention to avoid iron deficiency and anemia^30,31^. Therefore, it is advised that all pre—kindergarten children get a course of daily iron intake^30,32^. Consequently, this study attempts to ascertain and provide current insight into the quantity of iron containing foods consumption and associated characteristics among 6–23-month-old children in Ethiopia by using multilevel and spatial clustering analysis. According to this survey only 27.14% of children in Ethiopia aged 6–23 months consumed any of the required iron-rich food sources. This study has revealed a good consumption of iron containing foods, which is lower much lower than studies investigated in Australia (82.6%)^33^, Ireland (90%)^34^, Bangladesh (50%)^35^, and SSA (42.1%)^36^, conversely, the prevalence of good consumption of iron-rich foods in the present study is higher than the prevalence presented in Ethiopia (21.4%)^37^, and Madagascar (19.6%)^38^. A sociodemographic, cultural, or behavioral variation between the nations' approaches to child feeding practices could be a potential cause. Additionally, it could be that cultural differences in child feeding customs across the nation. Furthermore, since they are regarded as a luxury food rather than a necessary component of a child's diet on a daily basis, animal source foods are only consumed in Ethiopia during special social occasions^39^. In addition to this, the countries' dedication to mother and child health services, the sample size, and the analysis methodologies could also contribute to this disparity.

In the multilevel multivariable logistic regression model of iron foods intake among children aged 6–23 months in Ethiopia, regions, women's education levels, the number of under-five children in the family, health facility delivery, breast-feeding status, and the number of under-five children were statistically significant. According to this study, children whose mothers had completed their elementary and secondary educations consumed more iron than kids whose mothers had no formal education. The explanation could be that educated mothers understand the value of complementary feeding practices. Furthermore, their income level may have increased or they may have been able to secure their household's food security, and the empowerment of women increased as they engaged in education. These women may also have knowledge of the causes of iron deficiency anemia and ate iron-rich foods for their children^40^. Additionally, women who have received education may have a good impact on cultural child feeding customs. Educated women adhere to scientifically validated feeding practices, which are a reliable indicator of children receiving nutritious meals. Other similar studies show that children born from uneducated mother have a higher odd of developing anemia, taking low intake of iron rich foods compared to education children born from education mother, and vice versa^17,41,42^.

Compared to their contemporaries, children who were born in medical facilities had higher odds of consuming iron. Compared to those who give birth at home, mothers, who go to health facilities and gave birth there may have a better education level, urban residence, and a positive attitude towards childbirth by a health professional at health facilities. On the other hand, mothers who give birth at health facilities are given enough information by health professionals about what kind of food they should give to their children, breastfeeding, vaccination, seeking health treatment when they feel unhealthy. So, mothers can cultivate the habit of feeding and caring their children properly. Some mothers, even if iron rich foods are found in their homes, may not feed them to their children because of they do not understand the benefits. Similar studies have also investigated that iron intake, breastfeeding and other children feeding practices in several settings^43–45^.

According to this study, households with two or more children under the age of five had lower odds of consuming foods high in iron than those with one or fewer children. The possible reasons for the low odds of performance of mothers with two or more children under five in this study can be analyzed in several ways. This is one indication that these mothers do not use family planning, indication, these mothers may have low levels of educational attainment, no or low income and low self-determination or decision-making ability over the family. All these low education, multiple birth order, and wealth index can affect the consumption of iron rich foods^41,43^.

This finding demonstrated that iron intake among children residing in Afar and Somali regions was lower than those who live in the Addis Ababa regional metropolis. This can be explained by the fact that, compared to Addis Ababa, the economic activities in the Afar and Somalia regions are mostly controlled by the raising of cattle and the practice of pastoralist lifestyles, and agriculture is not common in these areas. Besides, since these two places did not have lush forests and water reservoirs, careers could not acquire wild fruit and fish, good sources of iron supplies^43,46^. Previous studies showed that vitamin and iron-rich foods were limited in the pastoral population, and meat and egg intake were low^47,48^. Due to a high rate of poverty and socioeconomic indicators that are far below national averages, the Ethiopian government designated the Afar, and Somali area as among developing regional states. Major obstacles to development include: inadequate management and implementation capacity, poor infrastructure, a lack of awareness and support for the high returns from investing in child-focused programming interventions, and a multitude of competing objectives. Lack of a platform to implement such initiatives, such as the National Nutrition Program and integrated Early Childhood Development, as well as a weak multi-sectoral approach in general might affect the healthy intake of iron to children and mothers^41,47,49^.

The use of weighted, nationally representative data with a sizable sample, which makes the study representative at the level of the nation, was its key strength. As a result, it has sufficient statistical power to generalize the findings from the research's setting to all children aged 6 to 23 during the study period. Since the information was gathered cross-sectionally through self-reported interviews, recollection errors and social desirability bias could have occurred. Despite the fact that data collectors are highly skilled and trained, deliberate or inadvertent barriers to communication may lead to interview bias. Furthermore, there were no other context- or culture-related characteristics available. Consequently, one should exercise caution when interpreting the study's findings.

## Conclusions

The practice of intake and supplementation of iron rich foods for infants under two age was declared minimal and continuing as one of the public burdens in Ethiopia, however compared to the EDHS 2011 and 2016, which were about 13% and 21.41%, respectively^29,37^, it has shown improvement. Maternal educational preparation, health facility delivery, breast feeding status, number of under five children in the family and regions were found statistically significant with intake of iron foods in Ethiopia. Mothers who are not engaged in formal education, and did not utilization family planning to their family should be counseled and encouraged. Similarly, institutional delivery should be encouraging and supported to all segment of the population in the country. All most all regions, especially such as Afar and Somali need a massive support and coordination of efforts among the federal and regional states. Unless it will difficult to halt the shortage of iron intake to these regions due to the nature of them under developed economy, fragile population structures and other culture and income related issues. Governments, healthcare providers, and programmers must build consistent awareness, financial aid, and technical assistance as well as promote awareness of those areas because iron intake consumption varies greatly between locations in Ethiopia. Since the government has been using and focusing on increasing the primary health care at all segments of the population, strengthening the primary health care utilization, improving the skill and habit of health extension workers by addressing the unreachable remote areas, may have a higher impact regarding the improvement of good consumption of iron-containing foods through their guiding and mentoring in Ethiopia.

### Declaration

We hereby certify that the research was conducted using our own methods, in accordance with all applicable norms and laws, and after registering for data access at http://www.dhsprogram.com. We tried to account for all potential dangers associated with this research, got the requisite ethics and safety approval (where applicable), and demanded that the people in charge of the programme for the demography and health survey uphold our commitments and the rights of the participants. This research adheres to the Helsinki Declaration.

## Data Availability

The datasets generated and/or analyzed during the current study are publicly available in the Ethiopian demographic and health survey data repository of 2019, from the (http://www.dhsprogram.com).
